# Distal tibial interosseous osteochondroma with impending fracture of fibula – a case report and review of literature

**DOI:** 10.1186/1757-1626-2-115

**Published:** 2009-02-02

**Authors:** Iftikhar H Wani, Siddhartha Sharma, Farid H Malik, Manjeet Singh, Irfan Shiekh, Abdul Q Salaria

**Affiliations:** 1 Postgraduate Boys Hostel Room No 215 B, Government Medical College Bakshi Nager, Jammu, Jammu and Kashmir, India; 2 Postgraduate Department of Orthopaedics, Government Medical College, Jammu, Jammu and Kashmir, India

## Abstract

Osteochondromas arising from the interosseous border of the distal tibia and involving distal fibula are uncommon. We present a 16 year old young boy with an impending fracture, erosion and weakness of the distal fibula, secondary to an osteochondroma arising from the distal tibia. Early excision of this deforming distal tibial osteochondroma avoided the future risk of pathological fracture of the distal fibula, ankle deformities and syndesmotic complications.

## Introduction

The largest group of benign bone tumours are the osteochondromas which are composed of spongy bone covered by a cartilaginous cap[1,2]. Osteochondromas arising from the interosseous border, deforming distal tibia and fibula and occurring prior to physeal fusion are well reported in the literature. Plastic deformation of tibia and fibula, mechanical blocking of joint motion, syndesmotic problems (synostosis or diastasis), varus or valgus deformities of the ankle and subsequent degenerative changes in the ankle joint are some of the documented complications in the neglected cases [3,4]. Prior to skeletal maturity, a pathological fracture usually occurs if the osteochondroma is pedunculated [5]. However, the progressive growth of a sessile lesion in the distal metaphyseal region of the leg can lead to pressure erosion and scalloping of the neighbouring bone and a fracture may possibly ensue [6,7].

## Case presentation

**Figure 1:**
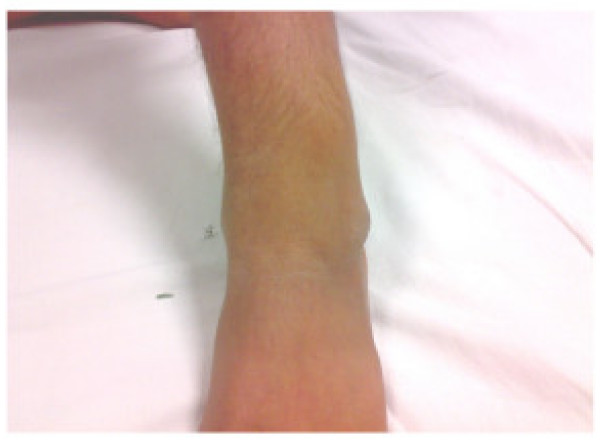
**Clinical photograph of a patient showing lesion over lateral aspect of ankle**.

**Figure 2:**
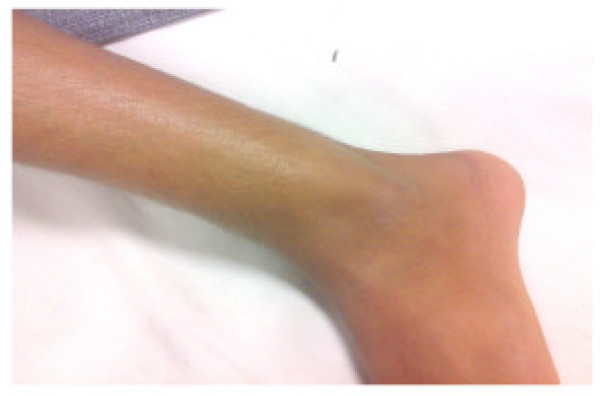
**Clinical photograph showing prominent swelling**.

A 16 year old male student of average built presented to us with progressively increasing swelling in the outer aspect of left ankle for last two years. Patient also gave history of pain off and on. There was no history of difficulty in walking or restriction of movements at ankle. There was a globular swelling measuring 6 cm × 5 cm over lateral aspect of ankle on examination (Fig. 1 and 2). It was bony hard in consistency, smooth with ill defined margins and non tender on palpation. There was no distal neurovascular deficit.

**Figure 3:**
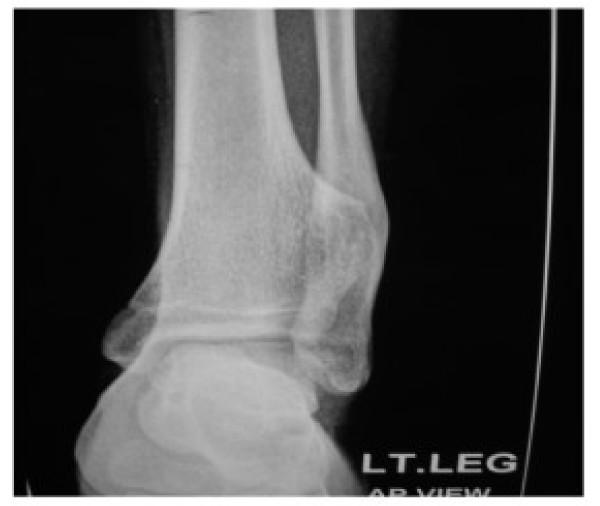
**AP radiograph of ankle of a patient showing well defined exostosis arising from interosseous border of tibia pushing fibula**.

**Figure 4:**
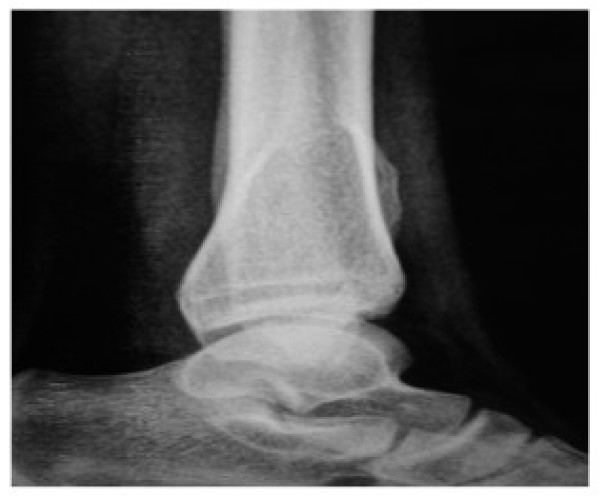
**Lateral Radiograph of ankle of a patient showing well defined exostosis arising from interosseous border of tibia pushing fibula**.

Patient was subjected to anteroposterior, lateral and oblique radiography of leg with ankle. Radiography revealed a well defined bony exostosis, arising from the interosseous border of distal tibial metaphysis with erosion and impending fracture of fibula on oblique view (Fig. 3 &4).

**Figure 5:**
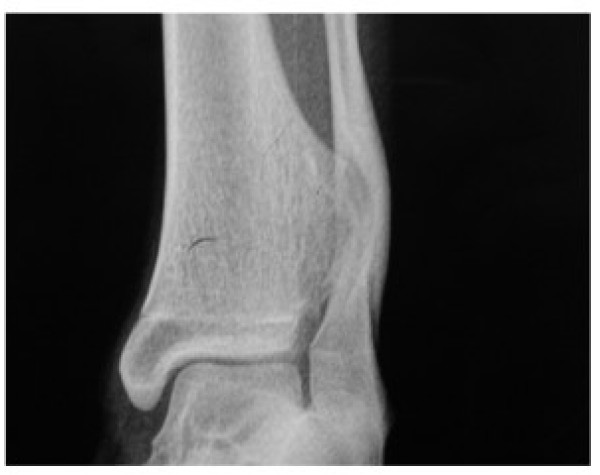
**Oblique view showing erosion and impending fracture of fibula secondary to tibial osteochondroma**.

**Figure 6:**
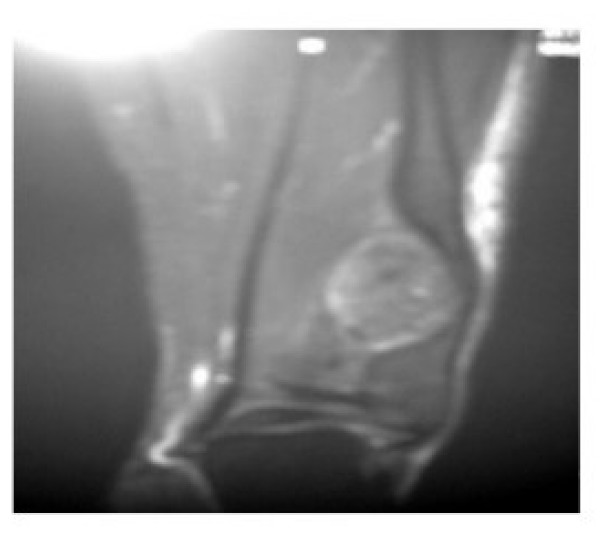
**MRI of the ankle showing osteochondroma with an uncalcified cartilaginous cap**.

The patient was initially put in an ankle foot orthosis. The nature and prognosis of the condition was discussed at length with the patient and his family and operative intervention was planned once an informed and written consent was obtained. An MRI scan was ordered once the decision was made to undertake operative intervention. It was consistent with a large, broad based benign osteochondroma arising from the lateral aspect of distal tibia with an uncalcified cartilagenous cap (Fig. 6). This led to a marked pressure erosion of the distal fibula, which was only 5 mm thick at the narrowest point (Fig. 5).

The patient underwent excision of the osteochondroma through an anterior approach without fibular osteotomy. Intra-operatively, the fibula was found to be quite thin and weak. However, its outer cortical shell was intact. The inferior tibio-fibular joint was stable. Histology confirmed the clinical diagnosis of osteochondroma with no malignant transformation. Post-operatively, the patient was mobilised, non-weight bearing in a below knee plaster, for four weeks. Further mobilisation was undertaken with a gradual transition from partial to full weight bearing. At one year follow-up, he had made a complete recovery with full return of ankle functions. The fibula had recovered the full thickness. There was no evidence of recurrence and he is still under follow up.

## Discussion

Osteochondromas are the most common benign bone tumours (40% of all benign, 10% of all primary skeletal tumours). They present most often in the second decade of life. The metaphyses of proximal tibia, distal femur, distal tibia, distal fibula, proximal femur and proximal humerus are the most commonly affected sites [3,4]. Osteochondromas arising from the tibial interosseous border and causing fibular erosion with imminent fractures after skeletal maturity are rare.

Osteochondromas usually follow a predictable course. The lesion slowly increases in size until physeal fusion. After skeletal maturity, the growth of this tumour slows down and eventually ceases in virtually all the cases. The main symptom is a mass or bony lump. Progressive enlargement of osteochondromata may cause nerve compression or skeletal deformity resulting in pressure symptoms. Malignant transformation to chondrosarcoma is rare (less than 1%) and should be suspected in the presence of increasing pain and sudden increase in the size of lesion in patients presenting after skeletal maturity [8].

The decision to treat distal tibial osteochondromas non-operatively carries the risk of persistence of symptoms and ankle deformity. Mirra (1989) reiterated the importance of complete resection of the cartilaginous cap to prevent recurrence [9]. In the previously published literature, anterior [5], posterior [7] and trans-fibular approach with fibular reconstruction [10] are described, although anterior approach without fibular osteotomy is associated with the least postoperative morbidity and was successfully used in this case.

## Conclusion

This case highlights the need for early excision of the osteochondromas deforming the distal aspect of tibia and fibula to prevent ankle deformities and syndesmotic complications and thereby obviates the need for complex reconstructive surgery.

## Consent

"Written informed consent was obtained from the patient for publication of this case report and accompanying images. A copy of the written consent is available for review by the Editor-in-Chief of this journal."

## Competing interests

The authors declare that they have no competing interests.

## Authors' contributions

"IHW and SS analyzed and interpreted the patient data regarding the disease. MS and SI discussed the case with radiology and pathology experts and formulated the treatment plan. AQS, FHM and SI performed the operative procedure. IHW was responsible for followup and prepared the manuscript. All authors read and approved the final manuscript."
